# Exposure to systemic antibiotics in outpatient care and the risk of multiple sclerosis

**DOI:** 10.1177/13524585231185045

**Published:** 2023-07-10

**Authors:** Jussi OT Sipilä, Matias Viitala, Arno Hänninen, Merja Soilu-Hänninen

**Affiliations:** Department of Neurology, Siun Sote, North Karelia Central Hospital, Joensuu, Finland and Clinical Neurosciences, University of Turku, Turku, Finland; StellarQ Ltd., Turku, Finland; Institute of Biomedicine, University of Turku and Clinical Microbiology, Turku University Hospital, Turku, Finland; Clinical Neurosciences, University of Turku and Neurocenter, Turku University Hospital, Turku, Finland

**Keywords:** Antimicrobial agent, autoimmune diseases, aetiology, microbiota, risk factor

## Abstract

**Background:**

Infections, early life exposures and the microbiome have been associated with the aetiology of multiple sclerosis (MS). Data on any possible roles of antibiotics is scarce and conflicting.

**Objective:**

The objective of this study was to investigate associations between outpatient systemic antibiotic exposure and the risk of MS in a nationwide case-control setting.

**Methods:**

Patients with MS were identified from the nation MS registry and their exposure to antibiotics was compared with that of persons without MS, provided by the national census authority. Antibiotic exposure was investigated using the national prescription database and analyzed by Anatomical Therapeutic Chemical (ATC) category.

**Results:**

Among the 1830 patients with MS and 12765 control persons, there were no associations between exposure to antibiotics in childhood (5–9 years) or adolescence (10–19 years) and the subsequent risk of MS. There was also no association between antibiotic exposure 1–6 years before disease onset and the risk of MS, save for exposure to fluoroquinolones in women (odds ratio: 1.28; 95% confidence interval: 1.03, 1.60; *p* = 0.028) which is probably associated with the increased infection burden in the MS prodrome.

**Conclusion:**

Use of systemic prescription antibiotics was not associated with subsequent MS risk.

## Introduction

Multiple sclerosis (MS) is caused by chronic dysregulation of immune homeostasis that results from complex interactions between genetic and environmental factors with an infection by the Epstein-Barr virus (EBV) almost a sine qua non.^1,2^ Other infections have also been reported to increase MS risk.^3–5^ However, it is unclear whether the drugs used to fight infections might also play a role. For example, many antibiotics disrupt the gut microbiota, which has crucial interactions with the human immune system and has been associated with MS.^6^ The few studies investigating associations between exposure to antibiotic and the risk of MS have mostly reported small cohorts, short observation periods and conflicting results.^7–9^

Studies on MS risk associated with migration have shown that exposures early in life are of paramount importance.^10–13^ It appears that there is no clear age cutoff for these and migration in adulthood also increases the risk, but there is a clear inverse correlation with age at migration and MS risk.^12,13^ Recent data on the evolution of the latitudinal gradient of MS suggest that early life environmental exposures are of paramount importance.^14^

We therefore devised a population-based case-control study to primarily investigate whether the use of systemic antibiotics in outpatient care before the age of 20 years was associated with the risk of MS in Finland. Considering the results of the recent Swedish study on hospital-diagnosed infections and the subsequent risk of MS,^5^ the risk was also assessed in subgroups of paediatric and adolescent antibiotic exposure. Second, considering the recently identified period of MS prodrome for at least 5 years prior to the first demyelinating event,^15^ we compared the antibiotic exposures of cases and controls during the 6-year period prior to the first MS symptoms of the patients.

## Materials and methods

Patients diagnosed with MS in 2016–2020 were identified from the national MS registry (*N* = 1809) and from North Karelia central hospital archives (N = 36).^16^ Seven age-, sex- and home municipality (at the time of MS diagnosis) matched controls were requested for every patient from the population registry held by the Digital and Population Data Services Agency (no control could be identified for 15 and one to six for 12 patients). The MS registry was used to exclude control persons who had a previous MS diagnosis (*n* = 39). The size of the final MS cohort was 1830 persons and the final control cohort 12765 persons (Table 1). Systemic antibiotics are available only on prescription in Finland. All purchase data of these (ATC codes J01-J05) were obtained from the Social Security Institution of Finland (SSIF) prescription database (data available only from the year 1995 onwards, excluding exposure at 0–4 years of age from the current analyses). Paediatric exposure was defined as antibiotic use at 5–9 years of age and adolescent exposure as use at 10–19 years of age. Considering the mean age of onset in MS,^17,18^ a sensitivity analysis was performed using only patients with MS onset ⩽30 years of age.

**Table 1.** Demographic and disease-related characteristics of the cohorts.

	Patients (*N* = 1830)	Controls (*N* = 12,765)
Sex		
Female, %	1279 (69.9%)	8915 (69.8%)
Male, %	551 (30.1%)	3850 (30.2%)
Age at index date (years)		
Mean (SD)	40 (13)	40 (13)
** Median (Q1, Q3)**	38 (30, 48)	38 (30, 48)
Age at MS onset (years)		
Mean (SD)	34 (11)	N.a.
Median (Q1, Q3)	33 (27, 41)	N.a.
Missing, %	474 (25.9%)	N.a.

N.a.: not applicable; Q: quartile.

### Statistical methods

Because prescription information was available from 1995 onwards, birth year-based exclusion for childhood and adolescence sub-analyses were made to have bias-free antibiotic exposure frequencies and odds ratios. Small cell rule (*n* < 5) was applied for frequency tables to ensure patient anonymity. For MS onset analyses, patients with missing onset year were excluded and the index year for controls were changed as corresponding onset year of the MS patients. Conditional logistic regression was used to get crude odds ratios and confidence intervals for antibiotic ATC groups stratifying models with matched case-control IDs. Controlling the false discovery rate (FDR), *p* values for each sub-analysis in Tables 2 and 3 were adjusted using the Benjamini and Hochberg method (1995) where smaller *p* values are penalized more than higher *p* values. All analyses were conducted using RStudio (Version 1.4.1103).

**Table 2.** Exposure to antibiotics by ATC group in multiple sclerosis (MS) patients and controls by sex and age of onset.

Exposure 1–6 years before MS onset	Female		Male		**All**	
	MS (*n* = 916)	Control (*n* = 6393)	MS (*n* = 386)	Control (*n* = 2697)	MS (*N* = 1302)	Control (*N* = 9090)
J01A (Tetracyclines)	283 (30.9%)	1892 (29.6%)	79 (20.5%)	603 (22.4%)	362 (27.8%)	2495 (27.4%)
J01C (Penicillin)	563 (61.5%)	3877 (60.6%)	169 (43.8%)	1202 (44.6%)	732 (56.2%)	5079 (55.9%)
J01D (Other betalactams)	386 (42.1%)	2647 (41.4%)	122 (31.6%)	883 (32.7%)	508 (39.0%)	3530 (38.8%)
J01E (Sulfonamide and Trimetoprim)	79 (8.6%)	465 (7.3%)	–	52 (1.9%)	–	517 (5.7%)
J01F (Macrolides, lincosamides, and streptogramines)	268 (29.3%)	1707 (26.7%)	80 (20.7%)	581 (21.5%)	348 (26.7%)	2288 (25.2%)
J01M (Kinolone derivates)	104 (11.4%)	582 (9.1%)	22 (5.7%)	190 (7.0%)	126 (9.7%)	772 (8.5%)
J02A (Systemic antifungals)	161 (17.6%)	1051 (16.4%)	8 (2.1%)	86 (3.2%)	169 (13.0%)	1137 (12.5%)
J05A (Systemic antivirals)	52 (5.7%)	410 (6.4%)	16 (4.1%)	76 (2.8%)	68 (5.2%)	486 (5.3%)
Exposure 1–6 years before MS onset (onset age ⩽ 30)	MS (*n* = 377)	Control (*n* = 2626)	MS (*n* = 134)	Control (*n* = 938)	MS (*N* = 511)	Control (*N* = 3564)
J01A (Tetracyclines)	109 (28.9%)	693 (26.4%)	26 (19.4%)	167 (17.8%)	135 (26.4%)	860 (24.1%)
J01C (Penicillin)	247 (65.5%)	1662 (63.3%)	72 (53.7%)	434 (46.3%)	319 (62.4%)	2096 (58.8%)
J01D (Other betalactams)	154 (40.8%)	1048 (39.9%)	50 (37.3%)	288 (30.7%)	204 (39.9%)	1336 (37.5%)
J01E (Sulfonamide and Trimetoprim)	35 (9.3%)	234 (8.9%)	–	18 (1.9%)	–	252 (7.1%)
J01F (Macrolides, lincosamides and streptogramines)	97 (25.7%)	634 (24.1%)	27 (20.1%)	171 (18.2%)	124 (24.3%)	805 (22.6%)
J01M (Kinolone derivates)	42 (11.1%)	226 (8.6%)	7 (5.2%)	46 (4.9%)	49 (9.6%)	272 (7.6%)
J02A (Systemic antifungals)	60 (15.9%)	397 (15.1%)	–	23 (2.5%)	–	420 (11.8%)
J05A (Systemic antivirals)	15 (4.0%)	133 (5.1%)	–	24 (2.6%)	–	157 (4.4%)

**Table 3.** Risk of multiple sclerosis associated with the use of different groups (ATC) of antibiotics in childhood (aged 5–9) and adolescence (aged 10–19).

	Exposure to antibiotics in childhood			Exposure to antibiotics in adolescence		
	OR	95% CI	*p* value	OR	95% CI	*p* value
Female						
J01A (Tetracyclines)	–	–	–	0.95	[0.74, 1.22]	0.69
J01C (Penicillin)	0.92	[0.70, 1.20]	0.55	1.17	[0.94, 1.44]	0.16
J01D (Other betalactams)	0.85	[0.65, 1.12]	0.26	1.06	[0.87, 1.28]	0.56
J01E (Sulfonamide and Trimetoprim)	0.91	[0.65, 1.27]	0.57	1.16	[0.88, 1.54]	0.29
J01F (Macrolides, lincosamides and streptogramines)	1.03	[0.80, 1.34]	0.81	1.06	[0.87, 1.29]	0.57
J01M (Kinolone derivates)	–	–	–	1.08	[0.72, 1.63]	0.70
J02A (Systemic antifungals)	–	–	–	1.15	[0.83, 1.58]	0.41
J05A (Systemic antivirals)	–	–	–	1.55	[0.95, 2.53]	0.082
Male						
J01A (Tetracyclines)	–	–	–	1.19	[0.81, 1.75]	0.37
J01C (Penicillin)	0.71	[0.46, 1.10]	0.13	1.15	[0.82, 1.60]	0.41
J01D (Other betalactams)	0.87	[0.56, 1.37]	0.56	0.99	[0.72, 1.36]	0.94
J01E (Sulfonamide and Trimetoprim)	1.11	[0.62, 1.99]	0.73	1.18	[0.67, 2.08]	0.57
J01F (Macrolides, lincosamides and streptogramines)	0.81	[0.52, 1.28]	0.37	1.13	[0.82, 1.57]	0.46
J01M (Kinolone derivates)	–	–	–	–	–	–
J02A (Systemic antifungals)	–	–	–	–	–	–
J05A (Systemic antivirals)	–	–	–	1.60	[0.60, 4.26]	0.35

CI: confidence interval; OR: crude odds ratio.

## Results

Exposure to antibiotics was common both in MS patients and controls, but there appeared to be differences in the use of different groups of antibiotics, especially according to age at exposure (Table 4). At the age of 5–9 years, exposure to most types of antibiotics seemed to have been more common in controls in both sexes, whereas MS patients had been exposed to most types of antibiotics slightly more often at the age of 10–19 years. Antibiotic exposure 1–6 years before MS onset appeared largely similar between patients and controls (Table 4), although in patients with MS onset ⩽30 years of age exposure to all antibacterials seemed slightly more common in the patients (Table 2). However, in the regression analyses, the risk of MS was not associated with exposure to any type of antibiotic in childhood or adolescence in either sex (Table 3). In the 6-year prodromal period before the first MS symptom, there were no association between exposure to antibiotics and the subsequent MS risk in men, whereas in women only exposure to kinolone derivatives associated with an increased MS risk (Table 5).

**Table 4.** Exposure to antibiotics by ATC group in multiple sclerosis patients and controls by sex, time-period, and age.

Exposure at any time before MS diagnosis	Female		Male		All	
	MS (*n* = 1279)	Control (*n* = 8915)	MS (*n* = 551)	Control (*n* = 3850)	MS (*N* = 1830)	Control (*N* = 12,765)
J01A (Tetracyclines)	735 (57.5 %)	5033 (56.5 %)	282 (51.2 %)	1839 (47.8 %)	1017 (55.6 %)	6872 (53.8 %)
J01C (Penicillin)	1139 (89.1 %)	7939 (89.1 %)	427 (77.5 %)	3112 (80.8 %)	1566 (85.6 %)	11051 (86.6 %)
J01D (Other betalactams)	1030 (80.5 %)	7077 (79.4 %)	399 (72.4 %)	2798 (72.7 %)	1429 (78.1 %)	9875 (77.4 %)
J01E (Sulfonamide and Trimetoprim)	427 (33.4 %)	2529 (28.4 %)	103 (18.7 %)	630 (16.4 %)	530 (29.0 %)	3159 (24.7 %)
J01F (Macrolides, lincosamides and streptogramines)	891 (69.7 %)	6011 (67.4 %)	319 (57.9 %)	2252 (58.5 %)	1210 (66.1 %)	8263 (64.7 %)
J01M (Kinolone derivates)	345 (27.0 %)	2091 (23.5 %)	114 (20.7 %)	706 (18.3 %)	459 (25.1 %)	2797 (21.9 %)
J02A (Systemic antifungals)	483 (37.8 %)	3111 (34.9 %)	48 (8.7 %)	365 (9.5 %)	531 (29.0 %)	3476 (27.2 %)
J05A (Systemic antivirals)	158 (12.4 %)	1142 (12.8 %)	44 (8.0 %)	260 (6.8 %)	202 (11.0 %)	1402 (11.0 %)
Exposure at 5–9 years of age	MS (*n* = 269)	Control (*n* = 1876)	MS (*n* = 96)	Control (*n* = 672)	MS (*N* = 365)	Control (*N* = 2548)
J01A (Tetracyclines)	–	–	–	–	–	–
J01 C (Penicillin)	172 (63.9 %)	1235 (65.8 %)	58 (60.4 %)	459 (68.3 %)	230 (63.0 %)	1694 (66.5 %)
J01D (Other betalactams)	84 (31.2 %)	653 (34.8 %)	31 (32.3 %)	238 (35.4 %)	115 (31.5 %)	891 (35.0 %)
J01E (Sulfonamide and Trimetoprim)	48 (17.8 %)	363 (19.3 %)	16 (16.7 %)	103 (15.3 %)	64 (17.5 %)	466 (18.3 %)
J01 F (Macrolides, lincosamides and streptogramines)	114 (42.4 %)	777 (41.4 %)	33 (34.4 %)	263 (39.1 %)	147 (40.3 %)	1040 (40.8 %)
J01M (Kinolone derivates)	–	–	–	–	–	–
J02A (Systemic antifungals)	–	–	–	–	–	–
J05A (Systemic antivirals)	–	–	–	–	–	–
Exposure at 10–19 years of age	MS (*n* = 477)	Control (*n* = 3325)	MS (*n* = 174)	Control (*n* = 1218)	MS (*N* = 651)	Control (*N* = 4543)
J01A (Tetracyclines)	94 (19.7 %)	676 (20.3 %)	38 (21.8 %)	231 (19.0 %)	132 (20.3 %)	907 (20.0 %)
J01C (Penicillin)	335 (70.2 %)	2228 (67.0 %)	112 (64.4 %)	745 (61.2 %)	447 (68.7 %)	2973 (65.4 %)
J01D (Other betalactams)	232 (48.6 %)	1569 (47.2 %)	82 (47.1 %)	578 (47.5 %)	314 (48.2 %)	2147 (47.3 %)
J01E (Sulfonamide and Trimetoprim)	66 (13.8 %)	405 (12.2 %)	15 (8.6 %)	90 (7.4 %)	81 (12.4 %)	495 (10.9 %)
J01F (Macrolides, lincosamides and streptogramines)	185 (38.8 %)	1250 (37.6 %)	69 (39.7 %)	448 (36.8 %)	254 (39.0 %)	1698 (37.4 %)
J01M (Kinolone derivates)	28 (5.9 %)	180 (5.4 %)	–	29 (2.4 %)	–	209 (4.6 %)
J02A (Systemic antifungals)	47 (9.9 %)	291 (8.8 %)	–	20 (1.6 %)	–	311 (6.8 %)
J05A (Systemic antivirals)	20 (4.2 %)	91 (2.7 %)	5 (2.9 %)	22 (1.8 %)	25 (3.8 %)	113 (2.5 %)

**Table 5.** Risk of multiple sclerosis (MS) associated with exposure to different groups (ATC) of antibiotics 1–6 years before MS onset.

	All patients with known age of onset			Patients with MS onset ⩽ 30 years of age		
	OR	95% CI	*p* value	OR	95% CI	*p* value
Female						
J01A (Tetracyclines)	1.07	[0.92, 1.25]	0.39	1.14	[0.90, 1.45]	0.28
J01C (Penicillin)	1.04	[0.90, 1.20]	0.62	1.11	[0.88, 1.40]	0.37
J01D (Other betalactams)	1.03	[0.89, 1.18]	0.70	1.03	[0.83, 1.29]	0.77
J01E (Sulfonamide and Trimetoprim)	1.21	[0.94, 1.55]	0.14	1.05	[0.72, 1.53]	0.79
J01F (Macrolides, lincosamides and streptogramines)	1.14	[0.98, 1.33]	0.10	1.09	[0.85, 1.40]	0.50
J01M (Kinolone derivates)	1.28	[1.03, 1.60]	0.028*	1.34	[0.94, 1.90]	0.11
J02A (Systemic antifungals)	1.09	[0.90, 1.31]	0.37	1.07	[0.79, 1.45]	0.66
J05A (Systemic antivirals)	0.88	[0.65, 1.19]	0.40	0.78	[0.45, 1.35]	0.37
Male						
J01A (Tetracyclines)	0.88	[0.67, 1.15]	0.36	1.11	[0.70, 1.76]	0.65
J01C (Penicillin)	0.96	[0.78, 1.20]	0.74	1.37	[0.94, 1.99]	0.098
J01D (Other betalactams)	0.95	[0.75, 1.19]	0.64	1.34	[0.92, 1.95]	0.13
J01E (Sulfonamide and Trimetoprim)	–	–	–	–	–	–
J01F (Macrolides, lincosamides and streptogramines)	0.95	[0.73, 1.24]	0.72	1.13	[0.72, 1.79]	0.59
J01M (Kinolone derivates)	0.79	[0.50, 1.25]	0.32	1.07	[0.47, 2.42]	0.87
J02A (Systemic antifungals)	0.64	[0.31, 1.34]	0.24	–	–	–
J05A (Systemic antivirals)	1.50	[0.86, 2.59]	0.15	–	–	–

OR: odds ratio; CI: confidence interval.

* BH corrected *p* values exceed 0.05.

## Discussion

We found no associations between MS and exposure to prescription antibiotics between 5 and 20 years of age. Furthermore, in the 6-year period before first MS symptoms, only women’s use of fluoroquinolones was associated with MS risk. Fluoroquinolones are recommended in outpatient care primarily for pneumonia and urinary tract infections (UTI) in Finland. According to the national Current Care guidelines, the diagnosis of a lower UTI does not have to be verified with a urine sample when the symptoms are typical. It is therefore likely that this association derives from the burden of infections, especially UTIs, around the time of MS diagnosis but possibly also from genitourinary symptoms associated with the MS prodrome.^19,20^ Indeed, it has been reported that the use of genitourinary and antimicrobial drugs is more common in the MS prodromal period compared with controls and that increased incidence of urinary tract disease is associated with the prodrome in females but not in males.^21,22^ However, considering the potential for microbiome disruption afforded by their wide efficacy spectrum, it cannot be excluded that fluoroquinolones may play a role in MS pathogenesis or onset.

Three previous studies have investigated associations between exposure to antibiotics and the risk of subsequent MS. The first was a prospective nested case-control study using the General Practice Research Database (GPRD) in the United Kingdom which reported no increased MS risk associated with overall antibiotic use or use of antibiotics against *C. pneumoniae*.^7^ Interestingly, the study also reported a decreased risk of developing a first attack of MS associated with the use of penicillins for over 2 weeks in the 3 years before the index date while penicillin use for 8–14 days was associated with an increased MS risk. Fluoroquinolones were not investigated separately, probably because they are seldom used for outpatients in the United Kingdom.^23^ Furthermore, the study included incident cases of MS recorded in 1993–2000 (*N* = 163), after which the diagnostic methods for MS have improved considerably.^24^ It is therefore unclear whether the results can be still considered relevant. The most recent study, conducted in Iran in 2013–2015, was slightly larger (547 incident MS cases) and reported that the overall use of antibiotics during 3 years before the index date was associated with a decreased risk of MS.^9^ The investigators adjusted the analyses for many currently known MS risk factors (age, sex, passive smoking, tobacco smoking, socioeconomic status, paternal education level during participants’ adolescence, years of education sunlight exposure, and history of depression), but data on the use of antibiotics was self-reported retrospectively and therefore prone to recall bias. Compared with our study, both of these studies were quite small and their generisability is unclear since the United Kingdom study only included patients in the GPRD, which has a very limited national coverage, and the Iranian study only included patients from Tehran.

A far larger (3259 MS cases) nationwide case-control study from Denmark used the Danish Multiple Sclerosis Registry (disease onset 1996–2008) and the National Prescription Database (data available for 1995–2008) reported that the use of almost any type of antibiotic was associated with an increased risk of MS with different antibiotics showing odds ratios of 1.08–1.83.^8^ Interestingly, this was lowest for quinolones with the 95% confidence interval crossing 1 only for these. This may be associated with the fact that fluoroquinolones are almost as seldom used for outpatients in Denmark as in the United Kingdom.^23^ The patients who have been prescribed quinolones are therefore likely to be highly selected. Importantly, the study defined no specific exposure period save ‘preceding the date of clinical onset of MS symptoms’ so the exposure may have happened 1–13 years before MS onset, obscuring any possible causal relationship and making a direct comparison to our results impossible. Unfortunately, both the Danish and our study lacked data on antibiotic dosage and number of treatment days. Nevertheless, the data currently available do not suggest a clear causal association between exposure to antibiotics and the subsequent MS risk.

Exposures in early life are clearly important in MS pathogenesis.^10–14^ There are no previous data concerning childhood or adolescence exposure to outpatient antibiotics and the subsequent risk of MS. Our data showed no such association. This is important, considering the fact that the gut microbiota has been associated with MS and in children it may be more malleable to environmental factors compared with adults.^6,25^ Our results therefore indicate that, according to the currently available data, MS risk is not a concern when prescribing antibiotics to children and adolescents. Interestingly, in our data the persons who subsequently developed MS appeared to have been exposed to antibiotics less often in childhood but more often in adolescence compared with controls. This suggests that the functional maturation of the immune system during puberty may have changed the susceptibility to infections treatable with antibiotics in persons prone to MS – or more severe infection-related symptoms, thus decreasing the everyday clinical threshold for prescribing.^26^ These findings are in line with the recent Swedish data reporting that any hospital-treated infection in adolescence, rather than in earlier childhood, increased the risk of a MS diagnosis from age 20 years.^5^

This study was based on retrospective registry data and therefore many data are missing. Most importantly, we do not have information about the clinical indications for the prescribed antibiotics. There were also not enough data available to perform separate analyses on exposure to antibiotics before 5 years of age or in patients with late-onset MS (⩾40 years of age). Moreover, exposure to a certain antibiotic was measured only as a binary since the medication data include only information on the drug purchases based on prescription but does not allow distinguishing between different prescriptions or make inferences about the number of distinct treatment courses. On the other hand, the prescription data are national and the coverage complete since systemic antibiotics are available in outpatient care only with prescription. Furthermore, the Finnish MS registry has a very high national coverage and completeness of the onset and diagnosis data.^16,27^ Finally, it is unclear how widely our results can be generalized considering the genetic peculiarity of the Finnish people and very high MS risk in the country.^28–30^

In conclusion, these data do not suggest an association between exposure to antibiotics and the subsequent risk of MS. Compared with controls, the persons who later develop MS seem less prone to infections treated with antibiotics in childhood but more susceptible to them in adolescence, suggesting that the maturation process of the immune system plays an important role.

## References

[1] Waubant E Lucas R Mowry E , et al. Environmental and genetic risk factors for MS: An integrated review. Ann Clin Transl Neurol 2019; 6(9): 1905–1922. 31392849 10.1002/acn3.50862 PMC6764632

[2] Bjornevik K Münz C Cohen JI , et al. Epstein-Barr virus as a leading cause of multiple sclerosis: Mechanisms and implications. Nat Rev Neurol 2023; 19(3): 160–171. 36759741 10.1038/s41582-023-00775-5

[3] Kurtzke JF . Epidemiology in multiple sclerosis: A pilgrim’s progress. Brain 2013; 136(Pt. 9): 2904–2917. 23983034 10.1093/brain/awt220

[4] Marrodan M Alessandro L Farez MF , et al. The role of infections in multiple sclerosis. Mult Scler 2019; 25: 891–901. 30638421 10.1177/1352458518823940

[5] Xu Y Smith KA Hiyoshi A , et al. Hospital-diagnosed infections before age 20 and risk of a subsequent multiple sclerosis diagnosis. Brain 2021; 144: 2390–2400. 33693538 10.1093/brain/awab100

[6] Correale J Hohlfeld R Baranzini SE . The role of the gut microbiota in multiple sclerosis. Nat Rev Neurol 2022; 18: 544–558. 35931825 10.1038/s41582-022-00697-8

[7] Alonso A Jick SS Jick H , et al. Antibiotic use and risk of multiple sclerosis. Am J Epidemiol 2006; 163: 997–1002. 16597708 10.1093/aje/kwj123

[8] Nørgaard M Nielsen RB Jacobsen JB , et al. Use of penicillin and other antibiotics and risk of multiple sclerosis: A population-based case-control study. Am J Epidemiol 2011; 174: 945–948. 21920946 10.1093/aje/kwr201

[9] Abdollahpour I Nedjat S Mansournia MA , et al. Infectious exposure, antibiotic use, and multiple sclerosis: A population-based incident case-control study. Acta Neurol Scand 2018; 138(4): 308–314. 29740825 10.1111/ane.12958

[10] Gale CR Martyn CN . Migrant studies in multiple sclerosis. Prog Neurobiol 1995; 47: 425–448. 8966212

[11] Wändell P Fredrikson S Carlsson AC , et al. Multiple sclerosis among first- and second-generation immigrant groups in Sweden. Acta Neurol Scand 2020; 142(4): 339–349. 32648932 10.1111/ane.13314

[12] Rotstein DL Marrie RA Maxwell C , et al. MS risk in immigrants in the McDonald era: A population-based study in Ontario, Canada. Neurology 2019; 93: e2203–e2215. 10.1212/WNL.0000000000008611 PMC6937488 31690681

[13] Munk Nielsen N Corn G Frisch M , et al. Multiple sclerosis among first- and second-generation immigrants in Denmark: A population-based cohort study. Brain 2019; 142: 1587–1597. 31081503 10.1093/brain/awz088

[14] Sabel CE Pearson JF Mason DF , et al. The latitude gradient for multiple sclerosis prevalence is established in the early life course. Brain 2021; 144: 2038–2046. 33704407 10.1093/brain/awab104

[15] Makhani N Tremlett H . The multiple sclerosis prodrome. Nat Rev Neurol 2021; 17: 515–521. 34155379 10.1038/s41582-021-00519-3 PMC8324569

[16] Laakso SM Viitala M Kuusisto H , et al. Multiple sclerosis in Finland 2018 – Data from the national register. Acta Neurol Scand 2019; 140(5): 303–311. 31271648 10.1111/ane.13145

[17] Romero-Pinel L Bau L Matas E , et al. The age at onset of relapsing-remitting multiple sclerosis has increased over the last five decades. Mult Scler Relat Disord 2022; 68: 104103. 36029708 10.1016/j.msard.2022.104103

[18] Prosperini L Lucchini M Ruggieri S , et al. Shift of multiple sclerosis onset towards older age. J Neurol Neurosurg Psychiatry 2022; 93: 1137. 10.1136/jnnp-2022-329049 35477891

[19] Castelo-Branco A Chiesa F Conte S , et al. Infections in patients with multiple sclerosis: A national cohort study in Sweden. Mult Scler Relat Disord 2020; 45: 102420. 32736217 10.1016/j.msard.2020.102420

[20] Tremlett H Munger KL Makhani N . The multiple sclerosis prodrome: Evidence to action. Front Neurol 2022; 12: 761408. 35173664 10.3389/fneur.2021.761408 PMC8841819

[21] Wijnands JM Zhu F Kingwell E , et al. Five years before multiple sclerosis onset: Phenotyping the prodrome. Mult Scler 2019; 25(8): 1092–1101. 29979093 10.1177/1352458518783662

[22] Perwieniec J Podwójcic K Maluchnik M , et al. Gender-related differences in prodromal multiple sclerosis characteristics: A 7-year observation study. J Clin Med 2021; 10: 3821. 34501269 10.3390/jcm10173821 PMC8432063

[23] Adriaenssens N Coenen S Versporten A , et al. European Surveillance of Antimicrobial Consumption (ESAC): Outpatient quinolone use in Europe (1997-2009). J Antimicrob Chemother 2011; 66(Suppl. 6): vi47–vi56. 10.1093/jac/dkr457 22096066

[24] Chataway J . Evolving diagnostic criteria for multiple sclerosis. Lancet Neurol 2018; 17: 118. 10.1016/S1474-4422(17)30463-5 29413308

[25] Derrien M Alvarez AS de Vos WM . The gut microbiota in the first decade of life. Trends Microbiol 2019; 27(12): 997–1010. 31474424 10.1016/j.tim.2019.08.001

[26] Ucciferri CC Dunn SE . Effect of puberty on the immune system: Relevance to multiple sclerosis. Front Pediatr 2022; 10: 1059083. 36533239 10.3389/fped.2022.1059083 PMC9755749

[27] Glaser A Stahmann A Meissner T , et al. Multiple sclerosis registries in Europe – An updated mapping survey. Mult Scler Relat Disord 2019; 27: 171–178. 30384204 10.1016/j.msard.2018.09.032

[28] Lao O Lu TT Nothnagel M , et al. Correlation between genetic and geographic structure in Europe. Curr Biol 2008; 18: 1241–1248. 18691889 10.1016/j.cub.2008.07.049

[29] Kurki MI Karjalainen J Palta P , et al. FinnGen provides genetic insights from a well-phenotyped isolated population. Nature 2023; 613: 508–518. 36653562 10.1038/s41586-022-05473-8 PMC9849126

[30] Pirttisalo AL Soilu-Hänninen M Sumelahti ML , et al. Changes in multiple sclerosis epidemiology in Finland over five decades. Acta Neurol Scand 2020; 142(3): 200–209. 32500607 10.1111/ane.13295

